# Biomass prediction and shoot growth characterization of single-staked yam plants using UAV imagery

**DOI:** 10.3389/fpls.2026.1776315

**Published:** 2026-04-01

**Authors:** Kohtaro Iseki, Ryo Matsumoto, Asrat Asfaw

**Affiliations:** 1 Japan International Research Center for Agricultural Sciences (JIRCAS), Tsukuba, Ibaraki, Japan; 2 International Institute of Tropical Agriculture, Ibadan, Oyo, Nigeria

**Keywords:** canopy structure, multi-angle UAV imagery, Richards growth model, shoot biomass, support vector regression, yam

## Abstract

This study presents an unmanned aerial vehicle (UAV)-based approach for estimating shoot biomass and characterizing growth patterns in single-staked white Guinea yams (*Dioscorea rotundata*). Multi-angle aerial images from nadir and oblique views were used to extract vegetation- and height-related indices that served as predictors in machine learning models. Support vector regression using combined-view imagery provided the highest prediction accuracy (R² = 0.79) and remained robust across growth stages, years, fertilizer treatments, and genotypes. Notably, the combined-view configuration outperformed single-view imaging, demonstrating the advantage of capturing complementary canopy-structure information in complex staked-vine canopies. Time-series biomass estimates enabled the fitting of genotype-specific Richards growth curves using Bayesian inference. Significant genotypic variations were observed in parameters associated with maximum biomass and early growth rate, whereas phenology-related parameters showed comparatively minimal differences. These parameter differences may reflect variation in canopy architecture and growth allocation among genotypes. Overall, this integrated workflow provides a scalable tool for nondestructive monitoring of yam growth dynamics and for summarizing biomass trajectories with interpretable parameters, supporting breeding efforts aimed at improving yam productivity and yield stability across diverse cultivation conditions.

## 1 Introduction

Yam (*Dioscorea* spp.) is a critical contributor to food security and supports the livelihoods of millions of individuals across tropical and subtropical regions, particularly in West Africa, where it constitutes a major staple crop. Although yams are culturally and economically important, the global average yield has remained unchanged for decades at approximately 7–8 t/ha (FAOSTAT, 2025). This lack of yield improvement notably contrasts with that of other major crops and presents a substantial challenge in the context of accelerating climate change and increasing food demands (Owusu Danquah et al., 2022).

To improve yam productivity, understanding the temporal dynamics of aboveground and belowground biomass development is fundamentally required. Tuber growth depends strongly on the translocation of carbon assimilates from the shoot, resulting in a strong correlation between aboveground biomass at the peak growth stage and tuber yield, although the strength of this relationship varies substantially among genotypes (Iseki and Matsumoto, 2020). Consequently, accurate and efficient assessment of shoot biomass is critical for genotype selection as well as for evaluating responses to environmental and management factors (Hgaza et al., 2010; Diby et al., 2011). However, current biomass measurements involve destructive sampling methods that are labor-intensive and impractical for large-scale or longitudinal studies, particularly in vegetatively propagated crops like yam that require substantial planting material (seed tubers), field space, and labor for staking and crop management.

Growth modeling approaches have been developed to address biomass estimation challenges in yams and other crops (Marcos et al., 2011; Cornet et al., 2022). These models provide interpretable parameters (e.g., maximum biomass, curvature/inflection timing, and senescence-related traits) that are useful for genotype-level comparisons. However, parameter estimates are often sensitive to observation frequency, measurement noise, and model structure; in particular, estimates can become unstable when sampling is infrequent or when consecutive observations contain highly similar information. In yams, obtaining reliable growth parameters for many genotypes remains difficult because of extensive intraspecies diversity, long growth cycles, and the necessity of preparing substantial quantities of seed tubers through clonal propagation. Moreover, the logistical burden of maintaining large numbers of replicates per genotype limits the scalability of traditional, destructive phenotyping. Therefore, nondestructive high-throughput approaches that can accommodate the complex morphology and propagation constraints of yams are needed.

Unmanned aerial vehicle (UAV)-based remote sensing has recently transformed plant phenotyping by providing a rapid, non-invasive approach for assessing crop growth, architecture, and vigor across multiple genotypes. Techniques using vegetation indices and canopy structure metrics have been successfully implemented in cereals, such as rice, maize, wheat, and soybean (Han et al., 2019; Atkinson Amorim et al., 2022; Ranđelović et al., 2023; Liao et al., 2025). However, these studies focused primarily on densely planted crops with uniform canopies, where top-down imagery sufficiently captures the horizontal expansion of the biomass. In contrast, crops with complex architectures, such as climbing or staked vines, pose additional challenges because vertical layering and self-shading reduce the visibility of lower canopy components. Moreover, commonly used spectral or projected-area features can saturate once the canopy becomes dense, making it difficult to discriminate among high-biomass plants (Escolà et al., 2023). To mitigate these issues, multi-view or oblique UAV imaging has been increasingly explored to provide side structural cues and improve the representation of canopy height and structure compared with nadir-only observations, enabling more reliable estimation of traits linked to biomass under heterogeneous canopy conditions (Lin et al., 2018).

For yams grown under staking systems, these challenges are amplified because individual plants form columnar canopies with strong vertical gradients. Although structure-from-motion (SfM)-derived point clouds can, in principle, support 3D reconstruction, field conditions (e.g., occlusion, limited side visibility, and wind-induced motion) often yield surface-biased and incomplete point clouds, making per-plant volumetric estimation unstable. These practical constraints motivate a field-ready approach that captures vertical canopy information without relying on full volumetric reconstruction at the individual-plant level. Consistent with this rationale, previous side-view spectral reflectance scanning studies have suggested that canopy height is a key determinant of shoot biomass in yams (Iseki and Matsumoto, 2019). In contrast, full volumetric 3D workflows—requiring dense point-cloud processing, extensive cleaning and classification, individual-plant 3D segmentation, and volume or mesh derivation—are computationally demanding and particularly sensitive to occlusion and motion, limiting their scalability in large breeding trials. Together, these observations highlight the need for practical UAV-based methods that robustly extract vertical canopy information for yam biomass phenotyping under field conditions.

In this study, we evaluated a combined approach integrating UAV-based remote sensing with machine learning, enabling scalable and nondestructive assessment under practical field conditions (Demir et al., 2024). We developed an image-based model to estimate the aboveground biomass of yams using UAV-derived oblique imagery. Using multi-angle aerial images, the proposed approach provides a simplified and cost-effective alternative to full 3D reconstruction while retaining critical height information. The model was validated across a range of yam genotypes to assess its utility in capturing growth variation. In addition, we used the estimated biomass data to determine genotype-specific growth parameters, such as maximum biomass, growth rate and curve chape, and the timing of senescence, thereby enabling the construction of parameterized growth curves tailored to individual genotypes. Overall, this study facilitates scalable phenotyping in yam and provides a foundation for broader applications to other vining crops or crops with complex, non-uniform canopies.

## 2 Materials and methods

### 2.1 Plant material and growth conditions

Twelve genotypes of white Guinea yam (*Dioscorea rotundata*), comprising breeding lines from the International Institute of Tropical Agriculture (IITA) and a widely cultivated local variety, were selected based on their contrasting shoot biomass and fertilizer responses (Matsumoto et al., 2021; **Supplementary Table 1**). Field cultivation was conducted at the experimental field of IITA in Ibadan, Nigeria (7°29′ N, 3°54′ E), during two consecutive cropping seasons: 2023–2024 and 2024–2025. The soil at the site was classified as sandy loam, moderately acidic (pH 5.8–6.1), and contained 4.3 g kg^−1^ organic carbon, 0.39 g kg^−1^ total nitrogen, and 3.1 mg kg^−1^ Bray-1 phosphate. Prior to planting, fields were plowed to ensure uniform soil conditions across plots.

To minimize the effects of different seed tuber sizes on early shoot growth (Iseki and Matsumoto, 2020), 100 g tuber blocks (setts) with a skin surface from which a shoot bud could emerge were cut from the center of normal-sized healthy tubers weighing approximately 1–2 kg. These setts were treated with a fungicide (a mixture of 70 g mancozeb and 75 mL chlorpyrifos in 10 L tap water) by dipping in the solution for 10 min, air-drying under shade for 24 h, and pre-sprouting for one month in plastic pots (12 cm diameter × 10 cm height) filled with sterilized sandy loam topsoil. The nursery soil had a pH of 7.6 and contained 2.0 g kg^−1^ organic carbon, 0.40 g kg^−1^ total nitrogen, and 3.8 mg kg^−1^ Bray-1 phosphate.

Transplantation was conducted on May 3, 2023 (DOY 123) and April 29, 2024 (DOY 120). Plants with adequate sprouts were selected for each genotype and transplanted individually with stakes into the field onto 40-cm-high ridges, spaced at 1 m × 1 m intervals, yielding a planting density of 10,000 plants ha^−1^. Each 3 × 3 m plot contained 16 plants. For each genotype, three replicate plots were established under fertilized conditions and three under unfertilized conditions, resulting in 72 plots arranged in a split-plot, randomized complete block design. Fertilizer treatment was assigned to the main plots, and genotype was assigned to the subplots. Fertilizer was applied three weeks after planting at a rate of 100 kg ha^−1^ using a compound formulation (N:P_2_O_5_:K_2_O = 14:23:14). Manual weeding was conducted throughout the growth period, and no additional fertilizer was applied.

### 2.2 Meteorological conditions

Meteorological data were recorded using an automatic weather station located at the experimental site. During the active growth period (June–December), both years exhibited the typical bimodal rainfall distribution characteristic of the region. Total precipitation measured 1227 mm in 2023 and 1086 mm in 2024. A short dry spell occurred in early August during both seasons. Daily average maximum and minimum temperatures measured 2 m above ground were 29.7 °C/22.9 °C and 30.7 °C/22.7 °C in 2023 and 2024, respectively. Daily solar radiation averaged 12.6 MJ m^−2^ in 2023 and 11.1 MJ m^−2^ in 2024.

### 2.3 Shoot sampling

Shoot biomass was destructively sampled at two growth stages, 60 d (August) and 120 d (October) after transplanting. In each plot, four adjacent plants were harvested, resulting in 1,152 sampled plants across two years (12 genotypes × 2 fertilizer treatments × 2 sampling times × 3 replications × 4 plants × 2 years). Shoots were separated from the tubers and oven-dried at 80 °C for 48 h to determine dry weight.

### 2.4 Aerial image acquisition and processing

**Figure 1:**
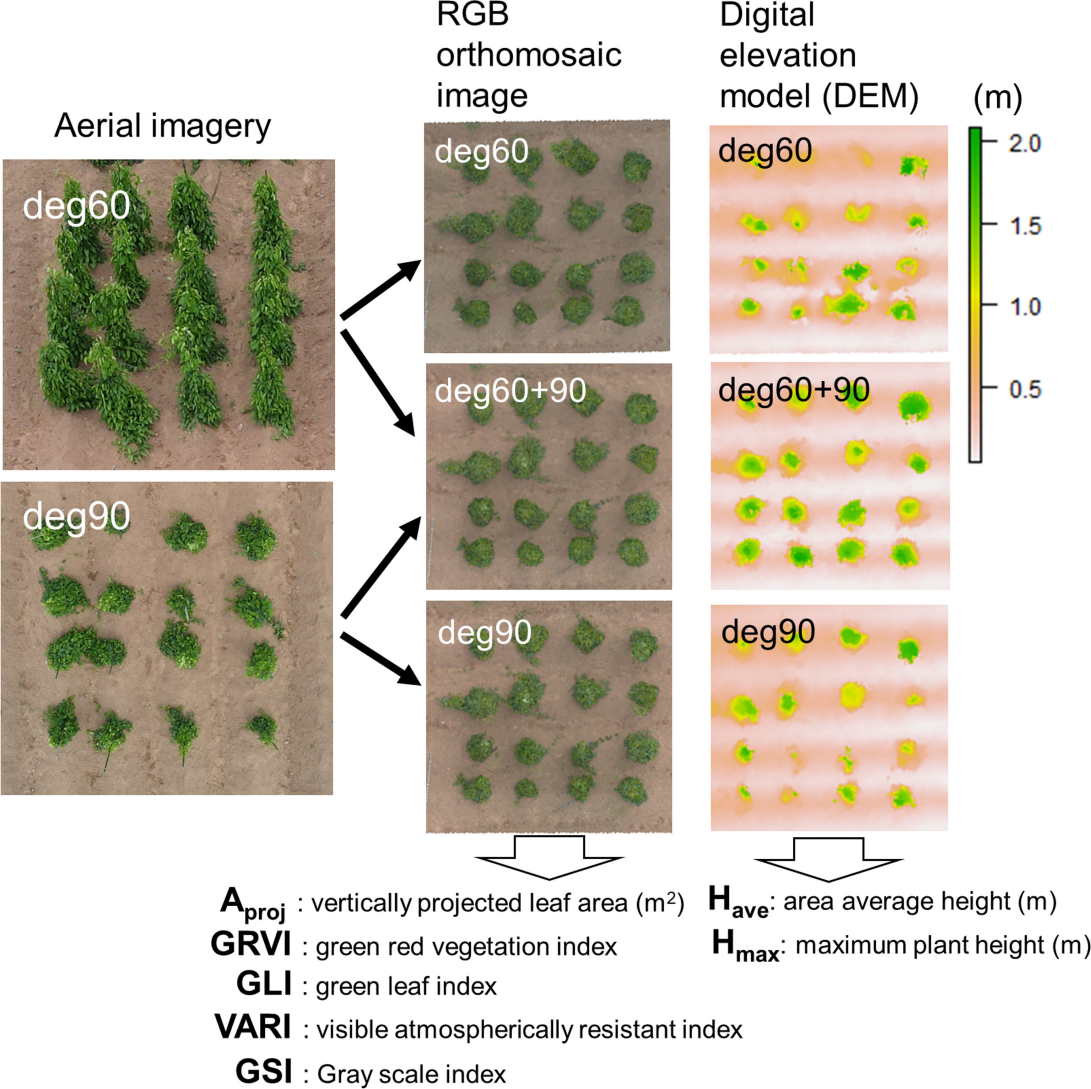
Extraction of vegetation- and height-related indices from nadir and oblique aerial imagery. RGB orthomosaic images and DEMs were generated separately for each image set: oblique (60°), nadir (90°), and the combined oblique and nadir views. Subsequently, vegetation and height indices were calculated independently for each dataset.

UAV-based imaging was conducted approximately one week prior to each biomass sampling date. Flights were performed over a 0.5 ha experimental field using a Mavic Air2 drone (DJI, Shenzhen, China). To evaluate the effect of imaging angle on biomass prediction accuracy, two sets of images were obtained: (1) oblique view at a 60°camera angle from 15 m altitude, and (2) nadir view at a 90° angle from 20 m altitude (**Figure 1**). These camera angles and flight altitudes were determined through preliminary testing to ensure adequate capture of the canopy structure of staked yam plants. Flights were conducted with 80% forward and lateral image overlap.

The captured RAW images were processed using MetaShape (version 2.1.1, Agisoft LLC, St. Petersburg, Russia) to generate orthomosaic images and digital elevation models (DEMs). Geometric corrections were performed using nine ground control points (GCPs) placed along the field borders and distributed evenly around the experimental area. All image georeferencing and clipping were performed using QGIS version 3.34.3. For each sampled plant, a 1 × 1 m polygon centered on the plant was created using custom R scripts, and the corresponding orthomosaic images and DEMs were extracted. This polygon size was sufficient to encompass the full canopy extent of all genotypes throughout the observation period.

### 2.5 Feature extraction from UAV data

Two categories of image-derived features were extracted: vegetation- and plant height-related indices (**Figure 1**). Projected canopy area (A_proj_) was calculated from the orthomosaic images using ImageJ version 1.8.0 (NIH, USA). Four vegetation indices were computed from RGB reflectance values: the Green–Red Vegetation Index (GRVI; Motohka et al., 2010), Green Leaf Index (GLI; Louhaichi et al., 2001), Visible Atmospherically Resistant Index (VARI; Gitelson et al., 2002), and Gray Scale Index (GSI; Hague et al., 2006). The GSI was derived from grayscale conversion, and its weighting coefficient (a) was optimized to maximize its correlation with A_proj_ (**Supplementary Figure 1**). A value of a = 0.635 was selected to calculate the GSI. These vegetation indices were selected as commonly used RGB-based indices that capture complementary variation in canopy greenness and brightness. As multispectral and hyperspectral sensors remain costly and are often impractical to deploy in local breeding programs, we focused on vegetation indices derived from standard RGB imagery acquired using a consumer-grade UAV. Using DEM data, two height-related indices, maximum plant height (H_max_) and mean plant height (H_ave_), were calculated as the vertical distance between the canopy surface and the local ground elevation. Seven explanatory variables were extracted from each UAV-derived image at oblique (60°), nadir (90°), and combined (60° and 90°) angles.

### 2.6 Biomass estimation modeling

To estimate shoot biomass from UAV-derived image features, we employed three machine learning algorithms, support vector regression (SVR), random forest (RF), extreme gradient boosting (XGB), and stepwise multiple linear regression to construct prediction models. A separate model was constructed for each set of variables extracted from images captured at oblique (60°), nadir (90°), and combined angles of 60° and 90°. The full dataset was randomly divided into 80% training and 20% testing subsets. Model construction used only the training subset, with 5-fold cross-validation employed to tune the hyperparameters and prevent overfitting. Predictive accuracy was assessed using the testing subset by comparing the predicted and observed shoot biomass values. Prior to model training, all explanatory variables were normalized to zero mean and unit variance. This standardization was performed to ensure consistent scaling across variables and to facilitate the comparison of Shapley additive explanation (SHAP) values across models.

For SVR, the models were trained using a radial basis function (RBF) kernel. A grid search was performed to determine the optimal combination of hyperparameters with the following candidate values: the cost parameter C set to 1, 5, 10, 50, and 100, and RBF kernel coefficient gamma, set to 0.01, 0.05, 0.1, 0.5, and 1. The best-performing model was selected based on the cross-validation root-mean-square error (RMSE). The RF models were constructed using 500 decision trees. The number of variables randomly selected at each split (mtry) was optimized using a grid search over the range of 1–4. Each tree was fully grown without pruning, and variable importance was calculated as the mean decrease in node impurity. The XGB models were developed using gradient boosting with the following hyperparameter ranges: learning rate set to 0.01, 0.1, and 0.3; maximum tree depth set to 3, 6, and 9; gamma set to 0, 0.1, and 0.2; subsampling rate set to 0.6, 0.8, and 1.0; minimum child weight set to 1, 3, and 5; and column subsampling rate set to 0.6, 0.8, and 1.0. Grid search and cross-validation were used to determine the best model configuration. Stepwise regression was performed using a bidirectional selection procedure based on the Akaike Information Criterion (AIC). Both forward selection and backward elimination were applied iteratively to identify the most parsimonious model without compromising predictive performance.

Model performance was evaluated using two metrics: the coefficient of determination (R^2^) and the RMSE. Robustness across growth stages, years, fertilizer treatments, and genotypes was assessed by *post hoc* grouping of the predictions from the held-out test subset. Specifically, after fitting the model on the training subset, we generated predictions for all samples in the test subset and then summarized prediction performance within each subgroup (year, sampling time, fertilizer treatment, and genotype). In addition, we employed SHAP analysis to interpret the model predictions and to understand the contribution of each predictor variable. SHAP values provide a unified measure of feature importance by attributing prediction differences to individual input variables in a game-theoretical framework (Wang et al., 2024). For stepwise regression, SHAP values were calculated only for the variables included in the final selected model. All modeling and analyses were performed in R (version 4.1.1) using the following packages: caret, e1071, randomForest, xgboost, MASS, and iml.

### 2.7 Growth curve fitting through Bayesian inference

The aim of growth-curve fitting was not to simulate mechanistic plant growth, but to provide a compact, descriptive parameterization of the UAV-estimated biomass time series. This parameterization enabled genotype-level comparisons of growth dynamics across the season using a small set of interpretable traits (e.g., maximum biomass, early growth curvature, and senescence timing), while quantifying uncertainty through posterior distributions.

An independent field trial was conducted in 2024 using the 12 genotypes described above. Eight plants per genotype were cultivated under non-fertilizer conditions, with three replicates in the same experimental field. UAV imagery was captured every two or three weeks from 2 to 18 weeks after transplantation, resulting in 11 time points per plant. Shoot biomass was estimated at each time point using the SVR model with combined oblique and nadir images, which had the highest predictive accuracy. The temporal biomass profiles of each plant were fitted using a composite nonlinear model incorporating both a logistic growth component of the Richards equation (Yin et al., 2003) and an exponential decay component (Pommerening and Muszta, 2016) (**Supplementary Figure 2**). The shoot biomass of genotype g at time t was expressed as follows:


$$f\left(\text{t};\;\theta_{g}\right)={\text{A}}_{g}{\left(1+{\text{ν}}_{g}\cdot {\text{e}}^{-{\text{k}}_{g}\left(\text{t}-{\text{t}}_{\text{i}.\text{g}}\right)}\right)}^{-\frac{1}{{\text{ν}}_{g}}}\times {\text{e}}^{-{\text{d}}_{g}\left(\text{t}-{\text{t}}_{\text{d}.\text{g}}\right)}$$

where *θ_g_* = (A_g_​, ν_g_​, k_g_​, t_i.g_​, d_g_​, t_d.g_​) denotes the genotype-specific parameters, A_g_ represents the asymptotic maximum biomass, ν_g_ controls the curvature of the logistic growth phase, k_g_ is the intrinsic growth‐rate constant that determines the characteristic timescale of biomass accumulation, and t_i.g_ is the inflection point at which the growth rate reaches its maximum. The exponential decay component includes the decay rate constant d_g_, which governs the characteristic timescale of senescence, and t_d.g_ represents the onset of decay.

To obtain the six parameters from the observed time series, we used a Gaussian likelihood (i.e., a Gaussian observation model) and estimated the parameters using a hierarchical Bayesian framework:


$$y_{gpr}\left(t\right)\sim Normal\left(f\left(t;\theta_{g}\right),\sigma_{obs}\right)$$

Let y_gpr_(t) denote the SVR-estimated shoot biomass for plant *p* of genotype *g* in replicate *r* at time *t*. σ_obs_ is the residual (observation) standard deviation capturing measurement and prediction noise in the estimated biomass, as well as model mismatch. Genotype-specific parameters were modeled hierarchically to borrow strength across genotypes:


$$\theta_{g}\sim Normal\left(\mu_{0},\Sigma_{0}\right),$$

with weakly informative prior on μ_0_ and $\sum_{0}$. μ_0_ and $\sum_{0}$ denote the population-level mean vector and variance–covariance matrix of the genotype-specific parameter vector *θ_g_*, describing the across-genotype average values and the among-genotype variability of the growth-curve parameters. The dataset comprised 3,168 observations (12 genotypes × 8 plants × 3 replicates × 11 time points).

Bayesian inference was performed using the No-U-Turn Sampler (NUTS) implemented in the Stan probabilistic programming language, accessed via the rstan package in R (version 4.1.1). The Markov Chain Monte Carlo (MCMC) procedure used four chains, each with 3,000 iterations, including 500 warm-up steps, yielding 10,000 posterior samples in total. Convergence was assessed using trace plots and the Gelman–Rubin statistic ( $\hat{R}$), with all $\hat{R}$ values< 1.1. The posterior distributions of all parameters were summarized and used to evaluate the variability and credible intervals across genotypes.

## 3 Results

### 3.1 Shoot biomass estimation models

**Figure 2:**
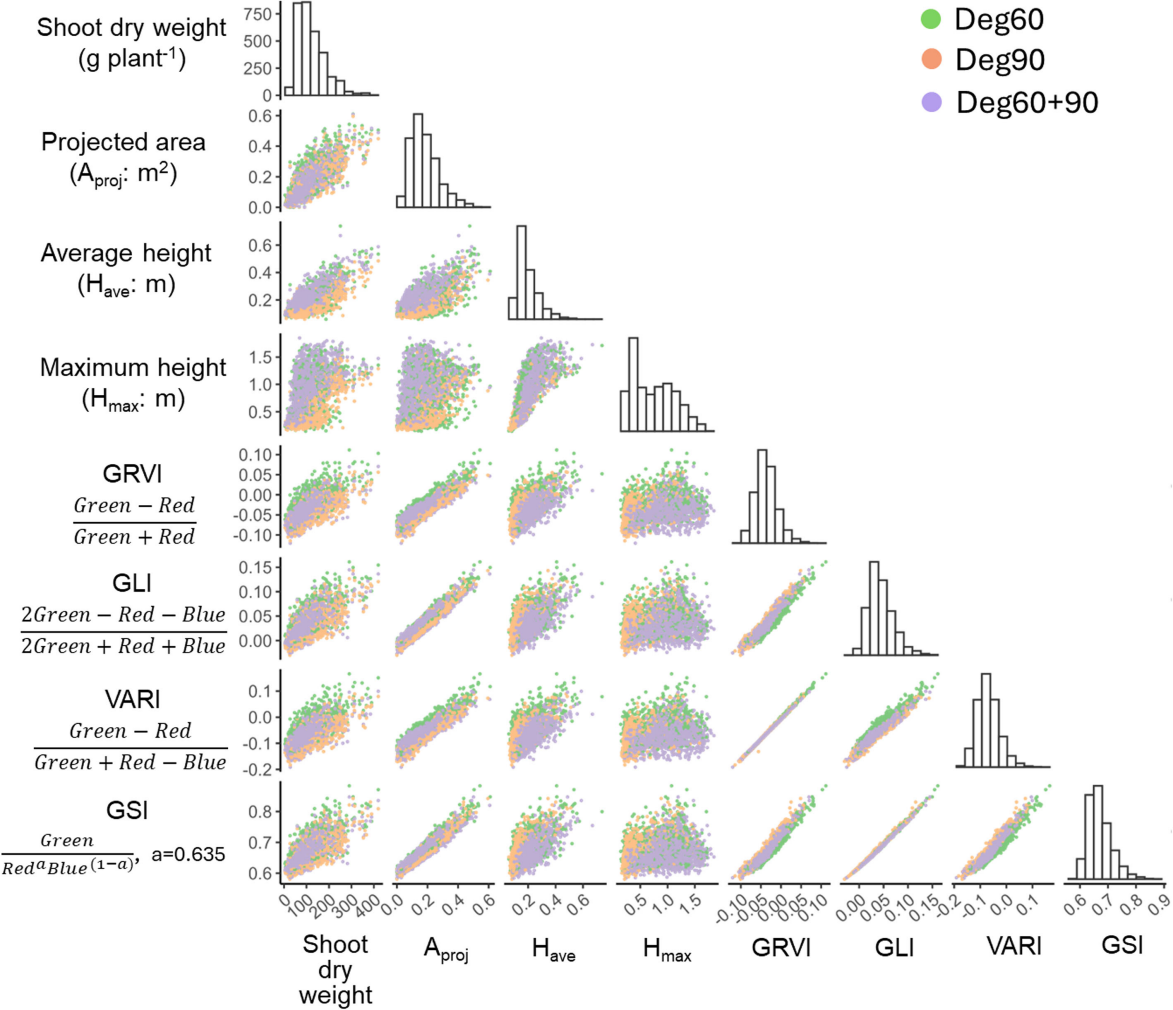
Correlation matrix among shoot dry weight and vegetation- and height-related indices. The dataset consists of 1,152 sampled plants derived from 12 genotypes × 2 fertilizer treatments × 2 sampling times × 3 replications × 4 plants × 2 years. Different colors indicate data obtained from different camera angles.

Wide distributions were observed in shoot dry weight, vegetation indices, and height-related indices used for model construction (**Figure 2**). The vegetation indices GRVI, GLI, VARI, and GSI were positively correlated with one another (correlation coefficient: r = 0.96–1.00, p< 0.01). Each of these indices showed a strong correlation with the projected canopy area (r = 0.87–0.96, p< 0.01), indicating that they effectively represented the green area in the image. The values of H_max_ were consistently higher than those of H_ave_, and substantial variation in H_max_ was observed among genotypes with similar H_ave_ values. The height indices calculated from the combined 60° and 90° images had higher average values than those derived from either oblique (60°) or nadir (90°) images alone. No single index was sufficient for shoot biomass estimation, although each index was positively correlated with shoot dry weight. The strongest and weakest correlations were observed for H_ave_ derived from 60° and 90° images and H_max_ derived from 60° images, with correlation coefficients of r = 0.84 and r = 0.55 (p< 0.01), respectively.

**Figure 3:**
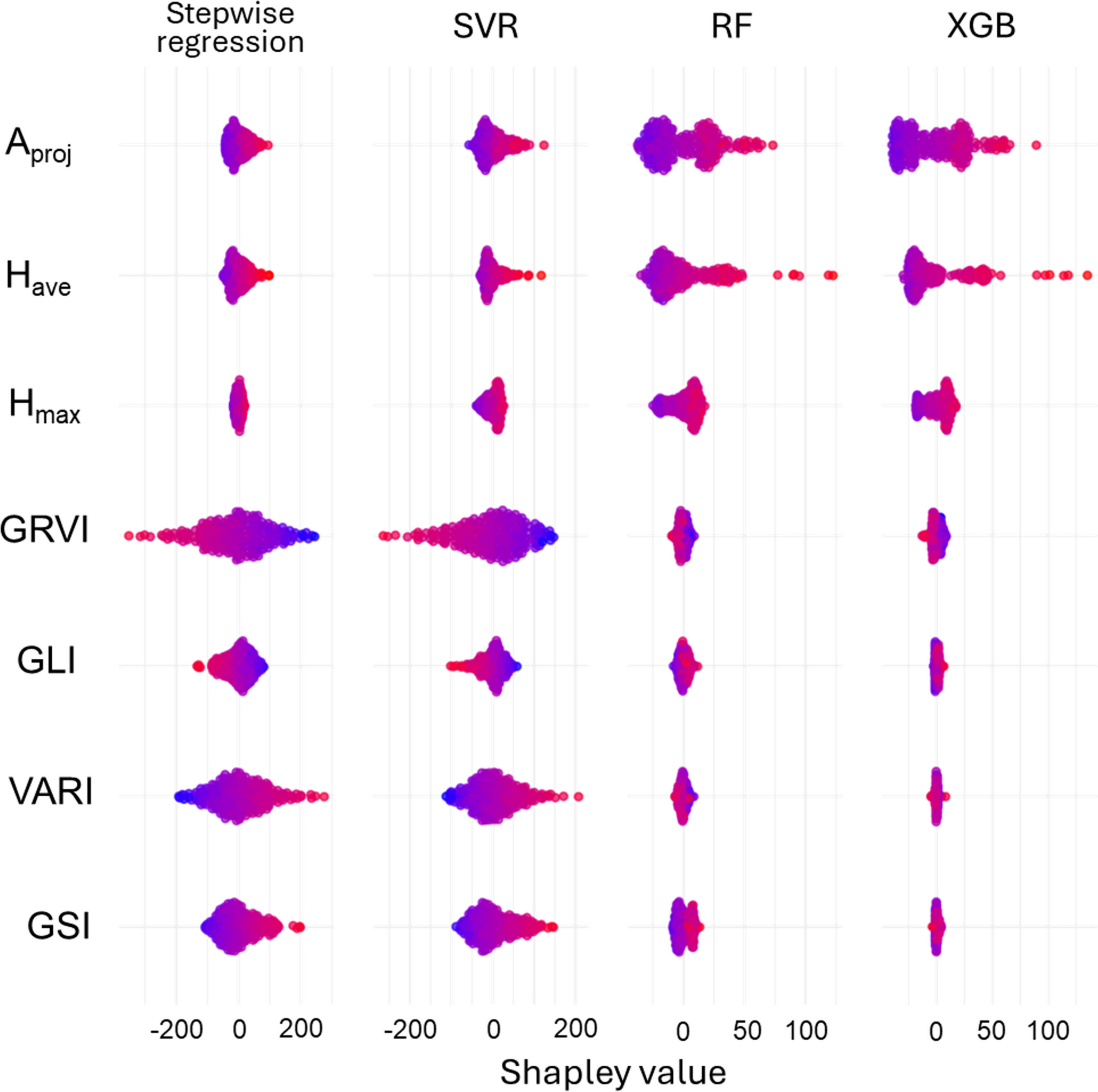
Shapley swarm plots showing feature-wise contributions to model predictions across four regression algorithms. Each point represents the SHAP value of a single test sample for a given feature. The vertical axis shows the Shapley value, indicating the direction and magnitude of each feature’s contribution to the prediction relative to the model baseline. Colors represent the original feature values for each test sample, mapped using a blue–red gradient (blue = low, red = high). Positive SHAP values indicate that a feature increases the predicted shoot dry weight, whereas negative values indicate a decreasing effect.

The dataset from images with different camera angles affected the estimation accuracy of shoot dry weight across the models. The dataset derived from images with combined camera angles consistently resulted in the highest estimation accuracy (highest R^2^ and lowest RMSE) for all regression algorithms (**Table 1**), whereas that derived from nadir images resulted in the lowest accuracy. Among the models, the best estimation accuracy was obtained using SVR, with R^2^ = 0.789 and RMSE = 29.9 g plant^-1^. Parameter contributions to biomass estimation were evaluated using SHAP values. Positive SHAP values indicated that a feature increased the predicted shoot dry weight, whereas negative values indicated a decreasing effect for a given prediction value. Across the algorithms, most predictions had positive SHAP values (red) for A_proj_ and H_ave_ (**Figure 3**), indicating that higher values of these two indices positively affected shoot biomass estimation. The vegetation indices showed different effects depending on the algorithms used. In the stepwise regression and SVR models, positive effects were observed for VARI and GSI, whereas negative effects were observed for GRVI and GLI; in contrast, these indices had minimal effects in the RF and XGB models.

**Table 1:** Shoot biomass estimation accuracy across four methods and three image sets with different aerial view angles.

Method	Angle of aerial image	RMSE	R^2^
Stepwise regression	deg60	35.9	0.728
	deg90	40.4	0.657
	deg60 + 90	30.6	0.773
Random forest regression	deg60	37.9	0.692
	deg90	40.3	0.658
	deg60 + 90	31.5	0.761
Support vector regression	deg60	37.0	0.716
	deg90	40.0	0.671
	deg60 + 90	29.9	0.789
XGBoost regression	deg60	37.5	0.701
	deg90	40.5	0.656
	deg60 + 90	31.8	0.756

Model validation was performed using a test dataset comprising 20% of 1,152 samples. deg60, deg90, and deg60 + 90 represent the datasets obtained from the oblique (60°), nadir (90°), and combined oblique–nadir aerial views, respectively.

**Figure 4:**
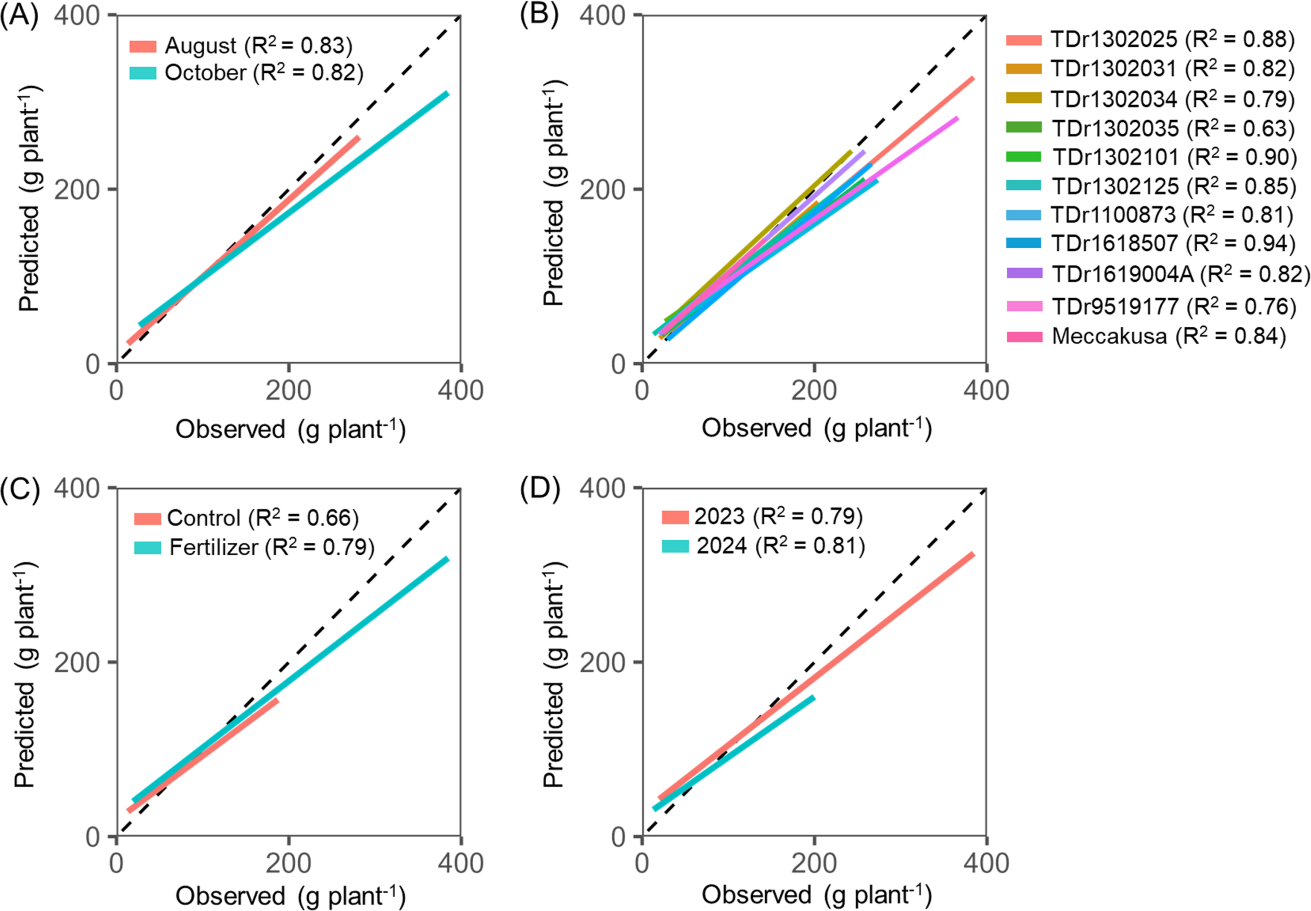
Biases in shoot biomass prediction by the best SVR model across **(A)** different sampling dates, **(B)** genotypes, **(C)** fertilizer treatments, and **(D)** cultivation years. Correlations were calculated using the 20% test subset of the total dataset of 1,152 sampled plants. The dotted line represents the 1:1 relationship between predicted and observed values.

The robustness of the best-performing SVR model was evaluated across different growth stages, genotypes, fertilizer treatments, and cultivation years (**Figure 4**). The estimation accuracy, expressed as the coefficient of determination, showed slight variation across growth stages and cultivation years. By contrast, relatively larger differences were observed among genotypes and fertilizer treatments, with coefficients ranging from 0.66 to 0.79 and from 0.63 to 0.94, respectively. However, the regression lines were similar among and within these factors, indicating that a single model was applicable for predictions across these conditions.

### 3.2 Growth characterization

**Figure 5:**
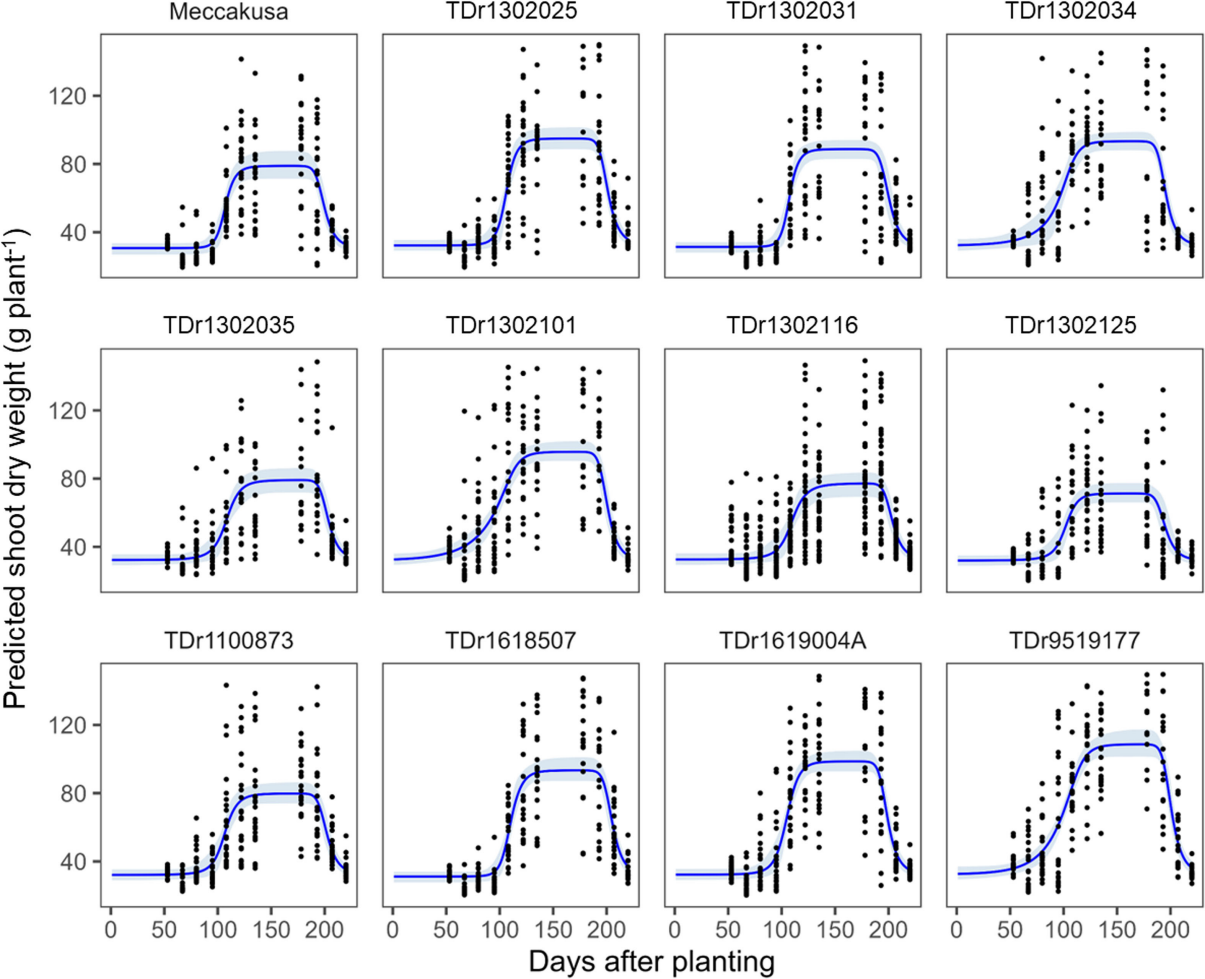
Time‐course distribution of predicted shoot biomass and genotypic variation in shoot growth curves fitted to the prediction outputs. Each time point includes 24 predictions derived from eight plants and three replications. Growth curves were fitted using posterior means and 95% credible intervals generated from 10,000 Markov chain Monte Carlo samples for each date.

Time-course predictions of shoot biomass for the 12 genotypes are shown in **Figure 5**. The considerable variation in shoot biomass at each time point indicates differences among plant replicates. Inter-plant variation was relatively small during the early and late growth periods, whereas it increased around the period of maximum shoot biomass. Despite this variability, the Bayesian inference showed good convergence of the growth-curve parameters (e.g., based on trace plots and $\hat{R}$), and the fitted growth curves captured the major temporal patterns of shoot biomass, including the initial increase, plateau, and subsequent decline.

**Figure 6:**
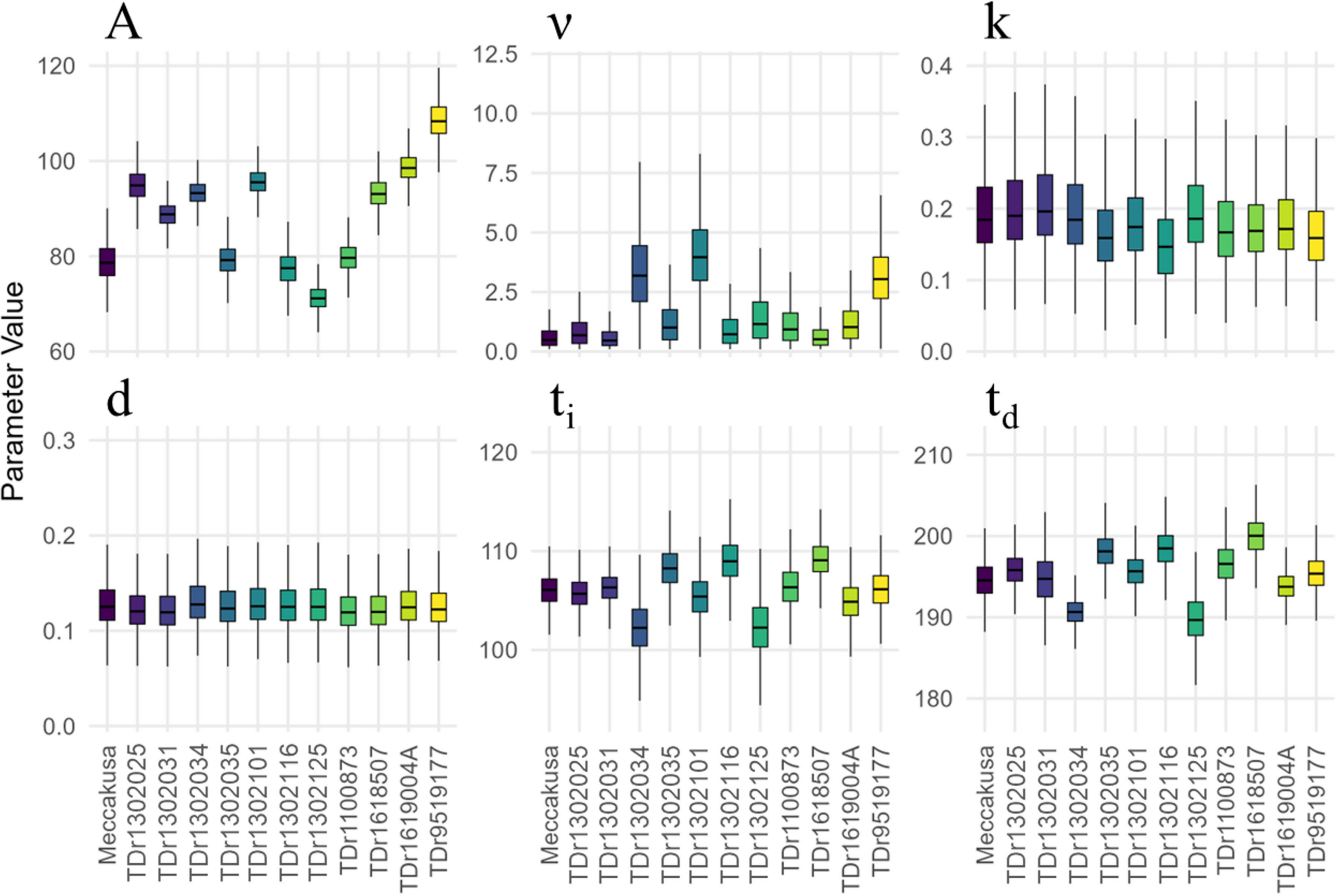
Posterior distributions of growth parameters obtained from the hierarchical Bayesian model. A, asymptotic maximum biomass (g plant^−1^); ν, growth curve shape factor (dimensionless); k, growth-rate constant (day^−1^); d, decay-rate constant (day^−1^); t_i_, time of maximum growth rate (DAP); t_d,_ decay onset date (DAP). Distributions were generated from 10,000 Markov chain Monte Carlo samples for each genotype. Horizontal lines within the boxes represent posterior medians. Box heights indicate the interquartile range, corresponding to the central 50% of samples, and whiskers show the approximate 99% posterior interval.

Curve fitting of the estimated biomass revealed apparent genotypic differences in growth dynamics, as reflected by Richards growth-curve parameters. Substantial variation was observed in A and ν, which represent the asymptotic maximum biomass and the shape (asymmetry) of the early growth phase, respectively (**Figure 6**). Seven genotypes (TDr1302025, TDr1302031, TDr1302034, TDr1302101, TDr1618507, TDr1619004A, and TDr9519177) exhibited particularly high A values. Specifically, three genotypes (TDr1302031, TDr1302101, and TDr9519177) also showed elevated ν values, indicating a slower initial acceleration of shoot biomass.

By contrast, the intrinsic growth rate constant, k, and decay rate constant, d, exhibited relatively small genotypic variability. However, genotypes with larger ν values tended to have smaller k values, consistent with a longer characteristic time to reach maximum biomass. The phenology-related parameters t_i_ and t_d_, representing the timing of the maximum growth rate and the onset of senescence, varied by less than 10 d (the ranges were DOY 100–110 for t_i_ and DOY 190–200 for t_d_) indicating broadly similar temporal patterns of growth and decay across genotypes.

## 4 Discussion

UAV-based biomass estimation facilitated the identification of apparent differences in aboveground growth characteristics among the staked yam genotypes. As yams exhibit distinct biological characteristics such as vegetative propagation, a long growth period, and low planting density, traditional destructive sampling enables the evaluation of only a minimal number of plants, making it challenging to infer representative growth patterns from limited samples. In contrast to previous studies that estimated shoot biomass using a destructive sampling of only two to three plants for a few genotypes (Hgaza et al., 2010; Cornet et al., 2022), our nondestructive UAV monitoring repeatedly evaluated 24 replicates for each of the 12 genotypes, resulting in the assessment of 3,168 plants across 11 time points. This extensive sampling enabled genotype-level growth characteristics to more accurately represent the true population mean, despite considerable variability among individual plants. Consequently, our results provide a foundation for UAV-based phenotyping as a more reliable and scalable framework for quantifying genotypic differences in yam growth characteristics and enhancing the precision of biomass estimation, consistent with advances reported in major cereals and legumes (Ranđelović et al., 2023; Yu et al., 2023; Shi et al., 2025).

Differences in biomass estimation accuracy among yam genotypes (**Figure 4**) may be related to canopy architecture and its interaction with UAV imaging geometry (Poley and McDermid, 2020). Traits such as leaf orientation, vertical layering, and the degree of exposed foliage can influence the visible vegetation surface captured by aerial imagery, as observed in maize (Khun et al., 2021). Accordingly, yam genotypes with erect or layered canopies present larger visible surfaces and may show stronger correlations with biomass, whereas differences in leaf density and within-canopy occlusion could contribute to biomass variation among plants with similar projected canopy areas (Pachakkil et al., 2021). Differences in estimation performance between fertilized and unfertilized treatments may reflect canopy-structural changes associated with nutrient status or nutrient-use efficiency (Matsumoto et al., 2022); however, we did not directly quantify these physiological mechanisms in this study. Notably, the fertilizer-related effects on accuracy were small compared with variation among plant replicates, suggesting that the proposed approach provides a reasonably robust characterization of yam shoot growth under the tested conditions.

Using both oblique and nadir UAV imagery improved biomass estimation for staked yam plants compared with using only oblique or nadir images. A key reason is that oblique views provide structural cues that complement nadir observations, particularly for vertically layered canopies. In our models, this complementarity was reflected in the height-related features derived from the DEMs (H_max_ and H_ave_). Oblique imagery can better expose stems, leaf angles, and lower canopy layers, which helps stabilize height estimation and reduces occlusion-related underrepresentation of vertical structure; when combined with nadir imagery, these effects likely improve the robustness of H_max_/H_ave_. This is consistent with previous findings that multi-angle imagery improves prediction across heterogeneous vegetation by capturing canopy density and mitigating saturation in purely 2D or spectral proxies (Sun et al., 2025), and that oblique imagery can provide more accurate representations of apparent plant surface area than mosaicked nadir imagery under radiometric degradation (Khun et al., 2021). Taken together, our results suggest that the combined-view approach enhances biomass prediction by improving the representation of canopy structure through the integration of height-related indices and RGB-based vegetation indices.

Although the combined dataset (60° + 90°) achieved the highest prediction accuracy (R² = 0.756–0.789), the improvement over the oblique-only configuration (R² = 0.716–0.728) was modest. Importantly, acquiring two viewing angles typically requires additional flight operations and increases data volume and processing time. For large-scale breeding programs in which throughput and simplicity are prioritized, oblique-only imaging may represent a pragmatic default that retains most of the achievable performance while minimizing operational burden. In contrast, when higher precision is required, the combined configuration is recommended despite the added effort. The optimal choice should therefore be guided by resource availability (flight time, labor, and computing capacity) and the level of precision required for decision-making.

In our shoot biomass models, vegetation indices, such as GRVI, GLI, VARI, and GSI were highly intercorrelated; however, their SHAP values occasionally indicated opposing contributions. This apparent inconsistency results from the fact that standard SHAP estimates marginal effects by perturbing individual features while holding the others fixed, which can generate unrealistic feature combinations that do not occur in the original UAV data. This instability under correlated predictors has been widely reported (Aas et al., 2021). Importantly, multicollinearity does not necessarily reduce predictive accuracy; ensemble learners such as random forest and gradient boosting can leverage redundant information and typically remain robust (Breiman, 2001; Friedman, 2001).

The consistently large SHAP values for projected canopy area (A_proj_) and mean canopy height (H_ave_) suggest that these structural traits are the primary drivers of biomass prediction. By contrast, the correlated vegetation indices likely provide complementary information that facilitates the stabilization of estimates across samples. We interpret A_proj_ and H_ave_ as biologically meaningful because canopy biomass is expected to scale with canopy extent and vertical development, which these variables directly represent under staking systems. Their consistently high contributions across years/stages/treatments, combined with GCP-based georeferencing and a fixed per-plant extraction polygon, suggests their importance is not solely an artifact of imaging geometry.

While genotype differences can be tested at individual time points, the curve-fitting approach summarizes the entire growth and yields interpretable growth-dynamic traits with uncertainty, facilitating genotype comparisons under irregular sampling and measurement noise. Variation in the Richards shape parameter ν provides insight into contrasting physiological strategies underlying yam growth. According to the framework presented by Chen et al. (2022), the parameter α, which is mathematically equivalent to ν, controls the curvature around the inflection point and indicates the degree of early growth limitation. In our data, genotypes with higher ν and lower k (e.g., TDr1302034, TDr1302101, and TDr9519177) showed slower initial shoot biomass accumulation. These patterns could arise from factors such as higher construction costs or lower early nitrogen acquisition or use efficiency, although these physiological mechanisms were not directly measured in this study. By contrast, genotypes with lower ν and higher k exhibited more rapid early canopy expansion, suggesting greater early vigor but potentially shorter periods of sustained biomass accumulation. Thus, the joint variation of ν and k provides a compact description of a trade-off between early vigor and delayed but steadier growth. Overall, the correspondence between ν and 𝛼 provides a useful conceptual framework for interpreting the observed genotypic differences, while recognizing that mechanistic validation would require additional trait measurements.

Genotypes with high A and k, including TDr1302025, TDr1302031, and TDr1302034, exhibited growth characterized by rapid early biomass gain and high estimated peak biomass. This parameter combination may indicate a strategy that couples early canopy development with sustained biomass accumulation later in the season. Similar growth patterns have been associated with high-yield plant types in other crops (Hunt and Cornelissen, 1997; Poorter et al., 2012). Their ability to achieve rapid initial growth without compromising final size suggests superior whole-plant carbon economics and highlights their potential value for yam improvement.

Finally, the limited genotypic variation observed in the phenology-related parameters t_i_ and t_d_ suggests that white guinea yam phenology is primarily influenced by planting date and seasonal climate rather than by genetic differences in photoperiod sensitivity, which is consistent with previous findings (Iseki et al., 2024). The onset of decay corresponded to the cessation of rainfall at the beginning of the dry season, when soil drying accelerated shoot senescence.

## 5 Conclusions

The combined use of nadir and oblique aerial images successfully captured the changes and variations in shoot biomass among genotypes throughout the growth period. The time series of biomass estimation further revealed apparent genotypic differences in the growth characteristics of staked yam plants. Rather than serving as a mechanistic explanation, the Richards model provided a useful descriptive framework for summarizing temporal growth patterns with interpretable parameters, thereby enabling robust comparisons of growth dynamics among genotypes. These trajectory-based traits can support future work linking shoot growth characteristics to tuber yield and can help inform breeding efforts aimed at improving yam productivity and production stability.

## Data Availability

The original contributions presented in the study are included in the article/**Supplementary Material**. Further inquiries can be directed to the corresponding author.
