# Supplementary material for: Biomass prediction and shoot growth characterization of single-staked yam plants using UAV imagery
Source: Front Plant Sci. 2026 Apr 1;17:1776315. doi: 10.3389/fpls.2026.1776315 (PMC13079368; doi:10.3389/fpls.2026.1776315)
Supplement: Supplementary file 1 [file DataSheet1.zip › Supplementary data sheet/Supplementary Figure 1.docx]

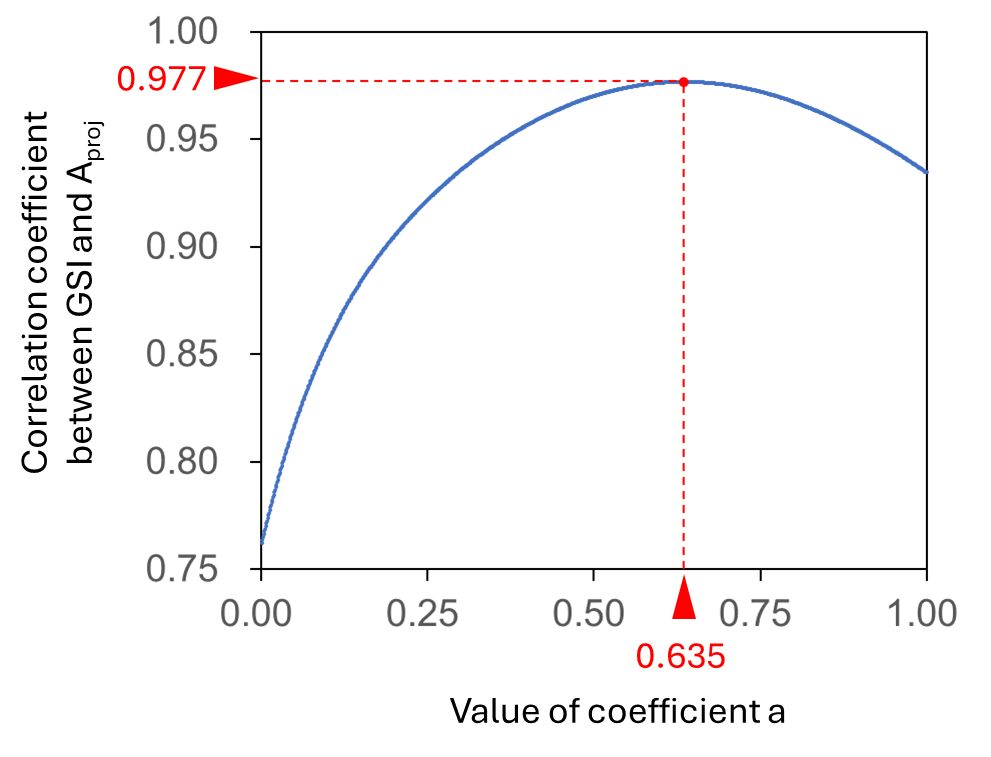


**Supplementary Figure 1.** Determination of the weighting coefficient (a) that maximizes the correlation between the Green–Senescence Index (GSI) and projected canopy area (A_proj_). The maximum correlation coefficient of 0.977 was obtained for a = 0.635.
