# Supplementary material for: Biomass prediction and shoot growth characterization of single-staked yam plants using UAV imagery
Source: Front Plant Sci. 2026 Apr 1;17:1776315. doi: 10.3389/fpls.2026.1776315 (PMC13079368; doi:10.3389/fpls.2026.1776315)
Supplement: Supplementary file 1 [file DataSheet1.zip › Supplementary data sheet/Supplementary Figure 2.docx]

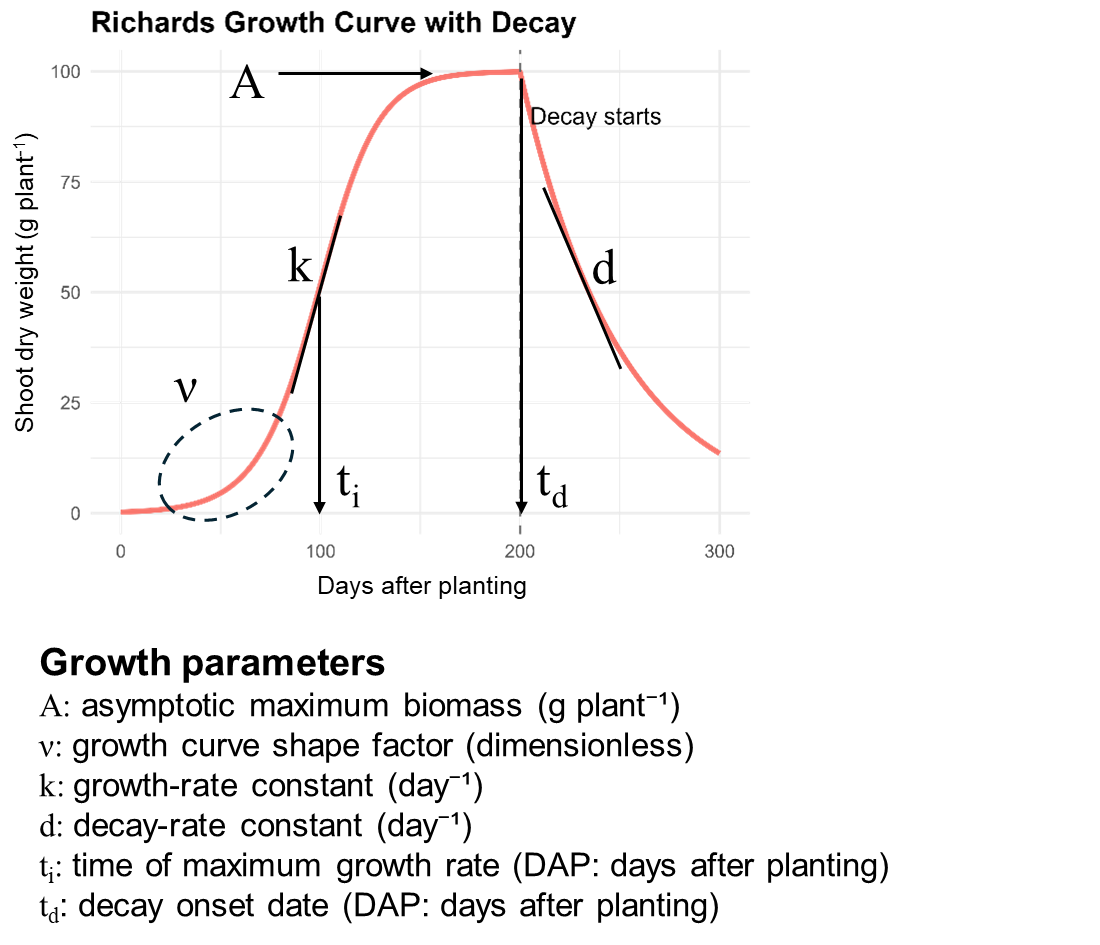


**Supplementary Figure 2.** Definition of parameters in the Richards growth curve, including those describing the decay phase.
