# Supplementary material for: Biomass prediction and shoot growth characterization of single-staked yam plants using UAV imagery
Source: Front Plant Sci. 2026 Apr 1;17:1776315. doi: 10.3389/fpls.2026.1776315 (PMC13079368; doi:10.3389/fpls.2026.1776315)
Supplement: Supplementary file 1 [file DataSheet1.zip › Supplementary data sheet/Supplementary Table 1.docx]

**Supplementary Table 1.** Twelve white guinea yam genotypes were examined.

| Name | Source | Description |
| --- | --- | --- |
| Meccakusa | Local variety |  |
| TDr1302025 | Breeding line | High fertilizer response |
| TDr1302031 | Breeding line |  |
| TDr1302034 | Breeding line | High fertilizer response |
| TDr1302035 | Breeding line |  |
| TDr1302101 | Breeding line | High fertilizer response |
| TDr1302116 | Breeding line |  |
| TDr1302125 | Breeding line | Low fertilizer response |
| TDr1100873 | Breeding line | High tuber yield |
| TDr1618507 | Breeding line | High tuber yield |
| TDr1619004A | Breeding line | High tuber yield |
| TDr9519177 | Breeding line | High tuber yield |

The fertilizer responses were obtained from Matsumoto et al. (2021).
